# FACTORS RELATED TO THE LIKELIHOOD OF AN ELECTRIC VEHICLE PURCHASE AMONG OLDER US ADULTS

**DOI:** 10.1093/geroni/igae098.1578

**Published:** 2024-12-31

**Authors:** Jonathon Vivoda, Renée St Louis

**Affiliations:** Miami University, Oxford, Ohio, United States; University of Michigan Transportation Research Institute, Ann Arbor, Michigan, United States

## Abstract

Widespread acceptance of electric vehicles (EVs) is important to meet global energy and pollution goals. Previous studies established that older adults are less likely than younger people to consider purchasing an EV, and common barriers to EV uptake among this group have been identified, but little else is known. The current study used two waves of data from the Pew Research Center’s American Trends Panel (April, 2021 and May, 2022), which contained EV-related questions. Only older adults who responded to both waves were included (n=2,016). Two ordinal logistic regression models were utilized to identify factors related to older adults’ likelihood of purchasing an EV (measured on a four-point scale), and to a change in likelihood between waves. Odds of higher likelihood of a future EV purchase were related to more education; Asian and Hispanic race (vs White); more liberal political ideology; stating that scientists understand causes of global climate change well; favoring the phase-out of gasoline vehicles; as well as stating that EVs are more fun to drive, more reliable, or better for the environment. Stating that EVs cost more was related to a lower likelihood, as were the reverse of the EV factors above. Only three factors were related to a change between waves in likelihood of purchasing an EV. Higher odds were related to identifying as a Democrat (vs Republican), high income, and having heard ‘a little’ about EVs vs ‘a lot.’ Findings, implications, and future research suggestions will also be discussed.

